# Management of Odontogenic Orbital Cellulitis

**Published:** 2011-08-25

**Authors:** FC DeCroos, JC Liao, NA Ramey, I Li

**Affiliations:** * Department of Ophthalmology Wills Eye Hospital, PhiladelphiaUSA; ** Department of Ophthalmology, Duke Eye Center, Durham, NC USA; *** Department of Family Medicine, Christiana Care Health System, Newark, DE USA

**Keywords:** orbital cellulitis, abscess, dental complications, tooth, infection

## Abstract

This work describes a series of patients with odontogenic orbital cellulitis (OOC), focusing on rate of abscess formation, need for surgical intervention, and imaging findings helpful for rapid diagnosis. Review of a current case and 22 patients with OOC from the scientific literature demonstrated periapical lucency as the most commonly (36.4%) reported finding on facial and orbital computerized tomography (CT) scan. Orbital abscess occurred in 72.7% of cases, and tooth extraction and/or abscess drainage was required in 95.5% of cases reviewed for control of infection. The current case presented with periapical lucency on CT scan, developed orbital abscess despite broad spectrum antibiotic therapy, and required multiple surgical interventions for disease resolution. Though our patient regained excellent vision, OOC often can result in severe vision loss. Periapical lucency on CT can help identify this atypical origin of cellulitis that is strongly associated with abscess formation and need for surgical intervention.

## Introduction

A 40–year–old gentleman with a history of hypertension presented to the emergency department with a 12 hour history of rapidly progressive facial pain, eyelid swelling, and visual loss suggestive of orbital cellulitis. This infection can lead to abscess formation, blindness, or death [1–4] and typically originates from sinus infections or cutaneous lesions. However, in this case presentation, the patient's infection originated from a very atypical location – a dental infection. This unusual origin for orbital cellulitis is associated with poor prognosis, frequent abscess formation, and need for multiple surgical interventions.

## Case presentation 

During detailed interview, the patient revealed that he had been well until 3 days prior, when he noted a right maxillary toothache. On review of systems, the patient endorsed diplopia, restricted right eye motility, and pain that worsened during eye movement. He was afebrile, with right periorbital and facial edema. The right eye itself demonstrated ptosis, proptosis, and slight exotropia. Visual acuity was 20/60, intraocular pressure was 16 mm Hg, and no afferent pupillary defect was detected. 

**Figure 1:**
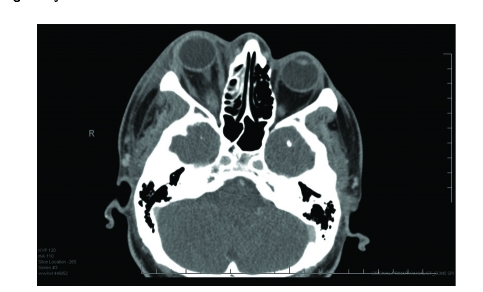
Axial CT scan (admission) shows right proptosis and facial soft tissue swelling

After admission to the hospital, laboratory studies revealed a white blood cell count of 8,500 with a manual differential that showed 13% bands. Computed tomography (CT) scanning demonstrated bilateral periapical lucency and inflammatory changes at the superior alveolar ridge consistent with dental infection, and breakdown of the right maxillary sinus base (Figure 1–Figure 3) suggestive of extension of infection from the molar roots into the maxillary sinus. Importantly, CT demonstrated right eye proptosis, orbital inflammation, and orbital emphysema adjacent to the right inferior rectus muscle, all consistent with orbital cellulitis (Figure 4). 

For this constellation of findings, the patient was treated with intravenous piperacillin/tazobactam. On the second hospital day, the right second and third molars were extracted. The patient's facial swelling, proptosis and ptosis improved, though eye pain and diplopia persisted. On the fourth hospital day the patient complained of increasing fever, chills, and worsening eye pain. The patient mounted a moderate leukocytosis of 17,000 white blood cells, visual acuity deteriorated to 20/200, and repeat CT scan showed a 24 x 18 x 21 mm orbital abscess indistinguishable from the right inferior rectus muscle (Figure 5). 

The patient underwent an urgent right orbitotomy for incision and drainage of the right orbital abscess. Wound culture was positive for anaerobic gram–positive cocci. On postoperative imaging no residual inflammation was identified. The patient was discharged to complete a 28–day course of piperacillin/tazobactam. By 3 weeks after discharge, there was complete resolution of proptosis and diplopia, and return of 20/20 vision in the right eye. 

**Figure 2:**
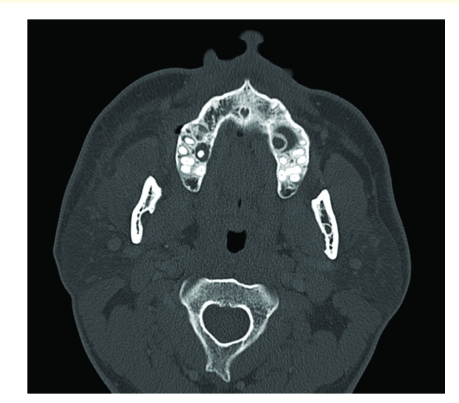
Axial CT scan (admission) shows bilateral periapical dental granulomas

**Figure 3:**
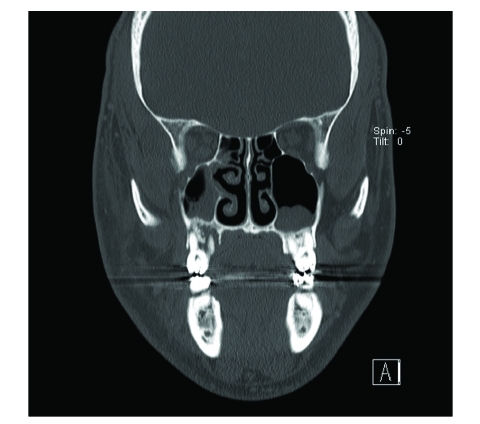
Coronal CT scan (admission) shows deterioration of right maxillary sinus floor

**Figure 4:**
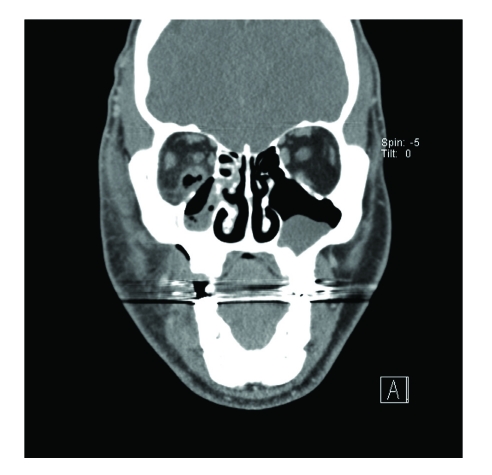
Coronal CT scan (admission) shows orbital emphysema continuous with the right inferior rectus and thickening of the adjacent maxillary sinus

**Figure 5:**
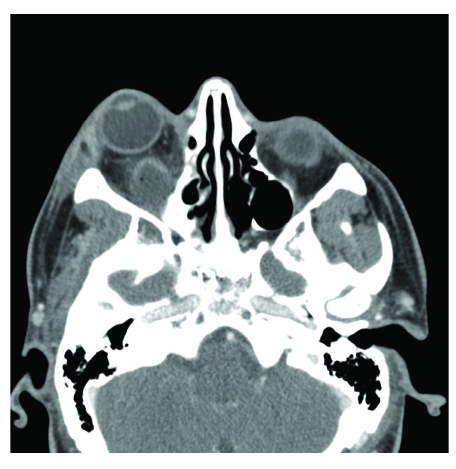
Repeat axial CT scan (hospital day 4) shows a right inferior orbital abscess

## Discussion

Our patient presented with an orbital cellulitis originating from a dental infection. Though he was treated with prompt tooth extraction and broad–spectrum antibiotics, his infection persisted and resulted in a sight–threatening orbital abscess.  Odontogenic orbital cellulitis (OOC) is a very rare clinical entity [1], but one that is commonly associated with abscess formation, need for surgery, and poor prognosis. In a prior series, 45.8% of patients with OOC had final vision of light perception or worse [5]. Our patient required serial imaging, broad–spectrum antibiotics, and multiple procedures to control his infection, consistent with the intensive care required to treat other cases of OOC in the literature (Table 1).

**Table 1:** Presentation, imaging, and management of cases of Odontogenic Orbital Cellulitis. (s/p = status post, POD # = postoperative day, I+D = Incision and Drainage, Extraction = tooth extraction.)

Study	Cases	Imaging Findings	Recent Dental History	Intervention Required
Caruso, 2006[7]	5	Periapical lucency x 5, maxillary sinus opacification x 5, Abscess 2/5	0/5	Extraction x 4, I+D x 1
Ngeow, 1999[12]	1	Maxillary sinus opacification, abscess	S/p root canal	None
Munoz–Guerra, 2006[9]	1	Maxillary sinus opacification	POD # 5 s/p extraction # 3	I+D
Allan, 1991[16]	1	Periapical lucency, abscess	s/p lost restoration (5 days)	I+D
Janakarajah, 1984[17]	1	Periapical lucency	Toothache	Extraction
Bullock, 1985[4]	4	Sinus opacification x 4, Abscess x 4	POD # 2, 2, 5, and 15 s/p extraction	I+D x 4
Thacker, 1995[10]	1	No sinus opacification, Abscess	POD # 3 s/p extraction	I+D
Zacharides, 2005[3]	1	Sinus opacification, Abscess	POD # 2 s/p extraction	I+D, revision on hospital day # 8
Miller,1995[18]	1	Sinus opacification, Caries	Toothache, POD # 1 s/p cleaning	Extraction, I+D
Poon, 2001[11]	1	Bilateral dilated superior ophthalmic veins	Dental caries one month prior	I+D
Henry, 1992[13]	1	Maxillary, ethmoid, and frontal sinus opacification, Abscess x 2	Toothache	Extraction, Sinus exploration, I+D
Kim, 2007[19]	1	Periapical lucency, Abscess	Temporal Pain	Extraction, I+D
Mehra, 1999[8]	1	Maxillary sinus air–fluid level	Displaced root tip	Extraction, Aspiration
Kiddee, 2010[14]	1	Abscess	Toothache	Extraction, I+D
Wysluch, 2009[15]	1	Abscess	Toothache	Extraction, I+D

Extension of odontogenic infection into the orbit can occur via a variety of pathways. Root apices are anatomically proximal to adjacent muscle, connective tissue, and sinuses. The most common route of spread is through the maxillary sinus into the inferior orbit via the inferior orbital fissure or defect in the orbital floor [6–8]. This was the most likely route in our patient. Less common paths include extension via the pterygopalatine regions [9,10], infection ascending from the canine fossa to the orbit [7], or retrograde spread through the ophthalmic vein [11]. In our patient, infection near the root apex of the third molar likely penetrated through the buccal cortical plate to the maxillary sinus, and then ascended via a preexisting orbital floor defect. The imaging studies were suggestive for such a route, and consistent with the history of toothache. 

In cases of OOC, a thorough history can be instrumental to rapid appropriate diagnosis. In 13 of 22 published cases reviewed, patients presented with recent history of dental surgery and 4 presented with history of toothache [12–15] (Table 1). This sampling may reflect selection bias of cases that were reported primarily in the oral surgery and dental literature. For example, of 5 patients with OOC in another series, none were initially suspected of having an odontogenic source for their illness [7]. This suspicion of dental origin is important, as OOC almost always required surgical intervention for disease resolution. In fact, of the 22 cases reviewed, only 1 did not require either a tooth extraction and/or abscess drainage for resolution of symptoms

Abscess formation is likewise a common complication of orbital extension of dental infection. Of the 22 cases reviewed, 16 required incision and drainage of subperiosteal or orbital abscess (Table 1). This rate is surprising when considering that orbital abscess has been reported as the rarest kind of abscess in the maxillofacial region with a prevalence of only 1.3% [1]. The rarity of OOC prevents determination of precise rates of abscess formation, but even with data aggregated from case reports one can conclude abscess formation is a frequent sequela. Finally, in a case of OOC where abscess is not initially noted, the physician must retain a high index of suspicion for subsequent abscess formation despite proper initial intervention and promising clinical improvement as in the case presentation. 

Imaging is helpful in verifying a dental etiology of orbital cellulitis and CT has been shown to be the modality of choice [7]. Periapical lucency was the most common (36.4%) CT finding associated with OOC in the cases reviewed (Table 1). Other findings include widening of the periodontal ligament space, sinus opacification, and pre-malar soft tissue swelling [7]. Orbital CT can also verify inflammation in the orbital cavity and lead to prompt identification of need for interventions like tooth extractions, sinus exploration, or abscess drainage. Our patient demonstrated abnormal periapical dental granulomas, sinus opacification, and orbital emphysema, allowing us to determine the most likely origin and route of spread of the infection. Later, imaging allowed us to localize and expeditiously drain an abscess that formed despite intravenous antibiotics. 

Odontogenic orbital cellulitis is a rare but serious infection with high risk of visual loss. This infection can necessitate intensive monitoring, serial imaging, multi–disciplinary care, and surgical intervention. As not all patients will present with a dental complaint, or after a recent dental procedure, the dental origin may remain unsuspected during the acute presentation. It is important for the practitioner to consider this rare source for orbital cellulitis, to have a low threshold for imaging suspicious cases, and to consider the frequent association of abscess formation with this infection. 
